# Distribution characteristics and influencing factors of carbon storage in *Populus* plantations with different stand ages in the Luxi Yellow River floodplain, China

**DOI:** 10.3389/fpls.2026.1764796

**Published:** 2026-03-04

**Authors:** Zhibao Wang, Xuehui Sun, Yuwei Guo, Chuanjie Zhou, Jing Liang, Haibing Wu, Cheng Huang, Xiangbin Gao, Yanyi Zhang

**Affiliations:** 1 College of Agriculture and Biology, Liaocheng University, Liaocheng, China; 2 School of Life Sciences, Shandong University, Qingdao, China; 3 Soil Research Institute, Shanghai Academy of Landscape Architecture Science and Planning, Shanghai, China; 4 Key Laboratory of National Forestry and Grassland Administration on Forest Ecosystem Protection and Restoration of Poyang Lake Watershed, College of Forestry, Jiangxi Agricultural University, Nanchang, China

**Keywords:** biomass, carbon storage, forest age, *Populus* planted forest, Yellow River floodplain

## Abstract

To explore the relationship between carbon storage and environmental factors in *Populus* plantations of different stand ages, and to reveal the carbon sequestration mechanisms of *Populus* plantations across different age classes, this study employed field surveys and laboratory analysis to investigate the distribution patterns and influencing factors of carbon storage in trunk-branch-leaf-root-soil systems of *Populus* plantations with different stand ages (10 y, 30 y, 40 y, 50 y) in the Luxi Yellow River floodplain. The results showed that the carbon storage in trunks, branches, and roots increased gradually with increasing stand age, while the carbon storage in leaves reached a maximum of 7.52 t·hm^2^ at 40 y, followed by a gradual decrease. Soil carbon storage increased consistently with stand age. Overall, the total carbon storage of *Populus* plantations across different age classes exhibited a linear increasing trend with advancing standage. Correlation analysis, principal component analysis, and structural equation modeling indicated that diameter at breast height (DBH), tree height (H), tree age (AGE), and stand density (SD) were the key factors affecting carbon storage in *Populus* plantations. The findings of this study can provide theoretical basis and technical support for enhancing carbon sequestration and sink capacity, as well as ecological restoration of *Populus* plantations in the Luxi Yellow River floodplain.

## 1 Introduction

The forest ecosystem is the largest carbon sink in terrestrial ecosystems, and its carbon sequestration accounts for two-thirds of the total carbon sequestration of terrestrial ecosystems (Ruehr et al., 2023). Besides, forests play an increasingly prominent role in increasing carbon sinks and slowing down the increase in atmospheric greenhouse gas concentrations (Marvin et al., 2023). Therefore, accurately assessing the carbon storage of forest ecosystems and its distribution pattern is of great significance for precisely evaluating and predicting the carbon storage of planted forest ecosystems and understanding the carbon cycle process (Keith et al., 2009; Ren et al., 2013; Vargas-Larreta et al., 2017; Tang et al., 2018).

Currently, research on the carbon storage of forest ecosystems mostly focuses on large-scale spatial estimations (Kauppi et al., 1992; Chen et al., 2019). Some scholars have studied the spatio-temporal distribution patterns of forest ecosystem carbon storage at the global scale (Pan et al., 2024), national scale (Ma et al., 2017; Sun and Liu, 2019), and provincial scale (Chen et al., 2019) respectively. Some scholars have explored the spatial distribution characteristics and change rules of forest carbon storage by integrating global forest resource inventory data and remote sensing data (Wang et al., 2018; Xu et al., 2022). Sun et al. (2020) found that with the increase in latitude, forest vegetation gradually decreased, forest carbon storage decreased, and soil carbon storage increased. Differences in climate conditions, forest types, and management measures are the reasons for the significant differences in forest carbon storage in Australia, North America, and Europe (Keith et al., 2009). The forest carbon storage in China ranges from 3.3 Pg to 11.5 Pg. Ciais et al. (2008) and Hudiburg et al. (2011) found through integrating forest resource inventory data and ecosystem models that the forest carbon storage in the United States and Europe increased significantly from the 1990s to the early 21st century. Currently, most studies on forest carbon storage mainly rely on continuous national forest resource inventory data and use the volume-source biomass method to estimate forest carbon storage (Cheng et al., 2023). Although the above-mentioned studies have relatively reliable data support, they all study forest carbon storage from a large-scale perspective. At the small-scale level, the distribution of carbon storage in various organs of forest plants and soil remains unclear. Therefore, research on the distribution pattern of forest carbon storage at the small-scale level needs to be strengthened.

With the implementation of major forestry ecological projects in China, the area of planted forests has ranked first globally, and their carbon sequestration and sink enhancement effects have been remarkable (Yao et al., 2024). At present, numerous Chinese scholars have conducted studies on the carbon sequestration and sink enhancement functions of planted forests. For instance, comprehensive investigations into the relationships between vegetation carbon storage and environmental factors across different regions have been carried out by Chen et al. (2015) and Ni et al. (2022), and the regional distribution patterns of forest carbon storage have been revealed thereby. Since carbon sequestration is achieved as trees absorb carbon dioxide from the atmosphere and store it in their organs (Li et al., 2019), the clarification of biomass allocation patterns among various tree organs is regarded as a key step for calculating forest carbon storage (Lv et al., 2024). In addition, the carbon sequestration capacity of planted forests is often influenced by the factor of stand age (Diao et al., 2022; Cheng et al., 2024). Studies have demonstrated that with the increase in stand age, the biomass of tree trunks and branches increases while the biomass allocated to leaves and roots decreases, which in turn exerts an impact on the distribution patterns of carbon storage in planted forests (Zhou et al., 2020; Lv et al., 2024). Previous studies have mostly focused on the estimation of forest carbon storage at the community scale, while insufficient attention has been paid to the carbon storage of different organs such as trunks, branches of varying diameters, leaves, and roots of different categories including coarse roots, fine roots and dead roots among different tree species in forests, as well as that of deep-root systems. Therefore, in-depth analysis of the distribution patterns of carbon storage in various plant organs within forests provides crucial guidance for the accurate assessment of the carbon sink capacity of planted forest ecosystems.

The Luxi Yellow River floodplain is formed by alluvial deposits following the overflow of the Yellow River, where ecological problems including soil salinization and desertification exist. To improve the local ecological environment, poplar plantations have been established to alleviate soil salinization and desertification (Zhu et al., 2024a). Specifically, the planting area of poplars in the Luxi Yellow River floodplain has reached 7.57×10^6^ hectares (Zhu et al., 2024b). Current research on poplar plantations in this region has mainly focused on their ecological adaptability (Thakur et al., 2021). Reports concerning the distribution patterns and influencing factors of carbon storage in the plantations of this plain remain limited, which results in the unclear understanding of the driving mechanisms affecting the carbon storage of the poplar plantation ecosystem. Therefore, in this study, the methods of field investigation and laboratory analysis were adopted to study the distribution pattern of carbon storage and its influencing factors in *Populus* planted forests with different ages (10 y, 30 y, 40 y, and 50 y) in the Jiucheng Forest Farm of Gaotang County, Shandong Province. This study mainly aimed to solve the following problems: 1) What is the distribution pattern of carbon storage in the trunk, branches, leaves, roots, and soil of *Populus* planted forests with different ages? 2) What are the key environmental factors affecting the distribution of carbon storage in the trunk, branches, leaves, roots, and soil of *Populus* planted forests with different ages? The research results can provide a scientific basis for improving the management strategies of *Populus* planted forests and enhancing the carbon sink function of *Populus* planted forest ecosystems.

## 2 Materials and methods

### 2.1 Study area

**Figure 1:**
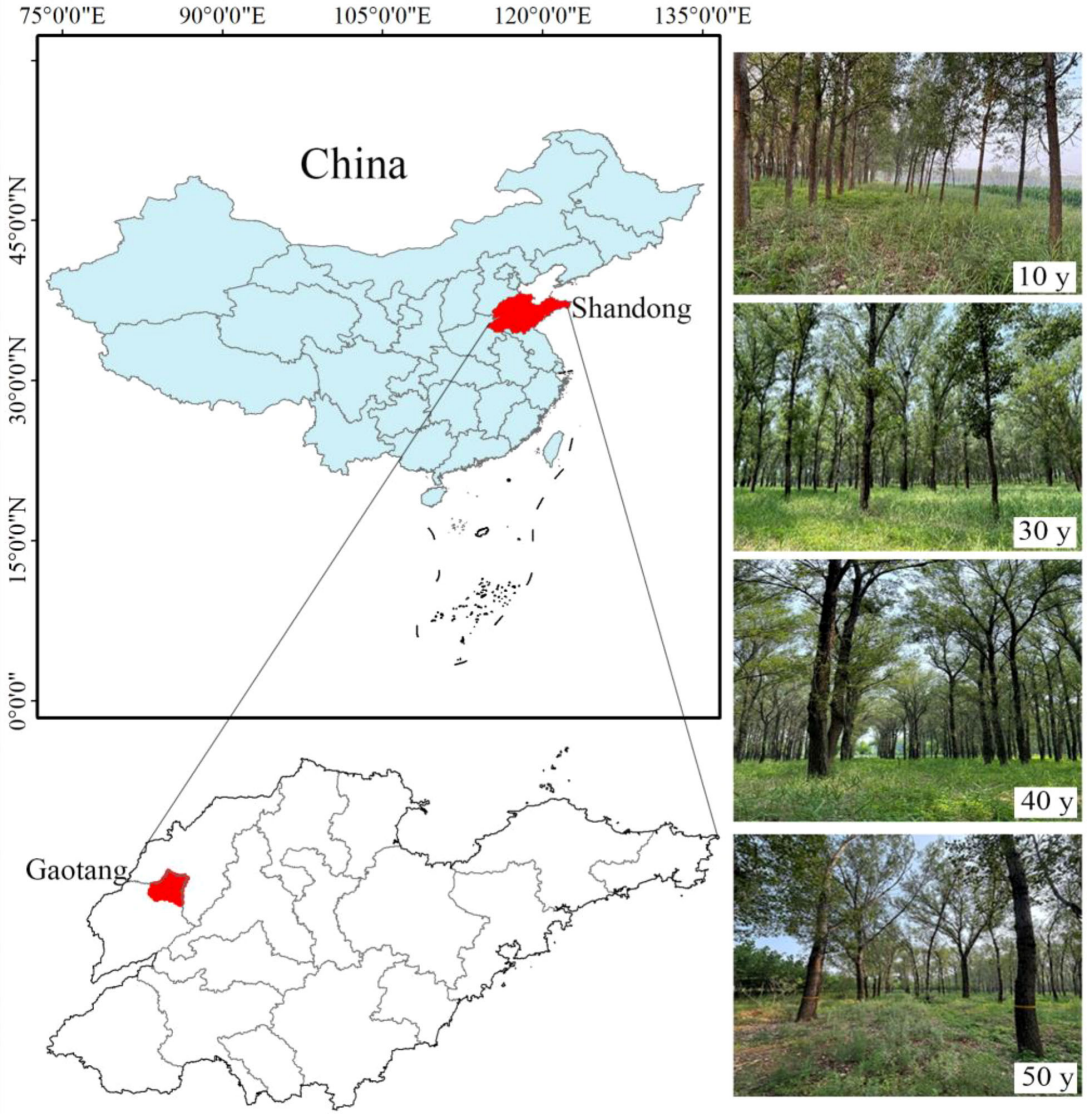
Study area.

The experimental site is located in the State-owned Jiucheng Forest Farm in Gaotang County, Liaocheng City, Shandong Province (35°47’ - 37°02’N, 115°16’ - 116°32’E, **Figure 1**). This area belongs to the warm-temperate semi-arid monsoon climate zone. The annual average temperature is 13.2 °C, the annual average wind speed is 3 m·s^-1^, the annual average precipitation is 287.1 - 954.0 mm, the annual average humidity is 75%, the annual sunshine duration is 2463 – 2741 h, and the frost-free period is about 193 - 201 d. The terrain of the experimental area is gentle. The soil parent material is the alluvium of the Yellow River, the soil type is sandy loam, and the vegetation type belongs to the warm-temperate deciduous broad-leaved forest. To control the problems of land desertification and soil erosion in the region, artificial forests have been planted in the forest farm, and the main planted varieties are *Populus* tomentosa and *Populus* simonii. The herbaceous plants are mainly self-sown plants, including *Phragmites australis*, *Humulus scandens*, *Setaria viridis*, *Bidens pilosa*, etc. The physical and chemical properties of the soil in the experimental site are shown in **Table 1**.

**Table 1:** Physical and chemical properties of soil in the test site.

Age/y	Soil bulk density/(g·cm^-3^)	Soil water content/(%)	Field capacity/(cm^3^·cm^-3^)	Soil porosity/(%)	pH	Electrical conductivity/(μs·cm^-1^)	Total carbon/(g·kg^-1^)	Total nitrogen/(g·kg^-1^)	Total phosphorus/(g·kg^-1^)	Organic matter content/(g·kg^-1^)	Available potassium/(mg·kg^-1^)
10	1.48 ± 0.01ab	4.16 ± 0.26b	0.25 ± 0.01a	47.15 ± 0.92a	8.35 ± 0.03a	105.01 ± 9.75a	2.12 ± 0.09e	0.32 ± 0.01b	0.46 ± 0b	3.59 ± 0.21e	43.57 ± 1b
30	1.43 ± 0.02c	4.73 ± 0.30ab	0.27 ± 0.01a	47.58 ± 0.61a	8.04 ± 0.04c	106.79 ± 14.32a	3.24 ± 0.17c	0.33 ± 0.01b	0.46 ± 0.01b	5.59 ± 0.3c	40.63 ± 1.86b
40	1.47 ± 0.01bc	3.37 ± 0.57c	0.27 ± 0.01a	48.08 ± 0.39a	8.21 ± 0.04b	117.06 ± 10.85a	3.80 ± 0.14b	0.37 ± 0.03a	0.45 ± 0.01b	7.70 ± 0.08b	51.10 ± 2.21a
50	1.45 ± 0.05bc	4.93 ± 0.58ab	0.27 ± 0.02a	47.93 ± 1.04a	8.21 ± 0.04b	103.15 ± 4.58a	7.14 ± 0.29a	0.39 ± 0.03a	0.45 ± 0b	12.31 ± 0.51a	52.30 ± 2.46a

Different lowercase letters indicated significant differences in the physical and chemical properties of different forest ages (*P*<0.05); the data in the table were the mean ± standard deviation.

### 2.2 Research methods

#### 2.2.1 Plot setting

Sampling was conducted on three separate occasions during July, August and September 2023. Four pure poplar plantations with different stand ages, namely 10 y, 30 y, 40 y and 50 y, were selected as the research objects in the Jiucheng Forest Farm of Gaotang County, with these plantations showing consistent growth status and site conditions. Three standard plots of 20 m × 20 m were set for each age group, and the interval between each standard plot was more than 20 m. A total of 12 plots were set. Then, each tree in the standard plots was measured, and factors such as the diameter at breast height, crown width, and tree height of the trees were measured (**Table 2**).

**Table 2:** Basic information of the sample.

Age/y	Plot area/m^2^	SD/(tree/hm^2^)	DBH/cm	H/m	W/m
10	400	916.66 ± 52.04	24.17 ± 0.76	21.33 ± 0.58	6.12 ± 0.31
30	400	450.00 ± 25.00	36.06 ± 1.03	32.67 ± 0.76	13.26 ± 0.30
40	400	391.67 ± 14.43	37.67 ± 0.76	35.09 ± 1.01	13.93 ± 1.10
50	400	341.67 ± 14.43	39.83 ± 1.04	37.83 ± 1.04	17.07 ± 1.69

Stand density, diameter at breast height, and tree height are expressed as mean ± standard deviation; SD, Stand density; DBH, Diameter at breast height; H, Height; W, Crown width.

#### 2.2.2 Sample collection

Sampling of arbor layer samples: Sampling was performed monthly from July to September 2023. In each sample plot, three poplar trees with basically consistent diameter at breast height, crown width and tree height were selected as sample trees for sampling. To avoid affecting the growth of *Populus* trees, after each tree measurement, a tree core was drilled at 1.3 m with an increment borer. Then, with a high-branch shear, one branch was cut from each of the four directions (east, west, south, and north) of the standard tree, and all the leaves were collected. The branches were divided into thick branches (≥ 10 cm), medium-sized branches (5 – 10 cm), thin branches (3 – 5 cm), and small branches (≤ 0.5 cm). Subsequently, using the “layer-by-layer cutting method”, the branches were sawed off at 0.5 m intervals, and a 10-cm-thick standard disc was sawed from the base of each section, and then the fresh weight of each section was weighed. To account for the spatial heterogeneity of root systems and ensure the scientific validity of root sampling, the “S”-shaped nine-point sampling method was adopted within each sample plot. A soil auger with a height of 10 cm and a diameter of 5 cm was sequentially driven into the soil profile to a depth of 100 cm. A total of 27 soil cores with intact root systems, each 100 cm in depth, were collected for each stand age group. Each soil core was divided into ten layers at depth intervals of 0 – 10 cm, 10 – 20 cm, 20 – 30 cm, 30 – 40 cm, 40 – 50 cm, 50 – 60 cm, 60 – 70 cm, 70 – 80 cm, 80 – 90 cm and 90 – 100 cm. Root samples from each layer were collected separately, placed into sealed plastic bags, labeled clearly and transported to the laboratory promptly for storage in a refrigerator at 4°C.

Soil Sample Collection: Sampling points were set at the center of each 20 m × 20 m standard plot. After removing the surface litter, a soil profile was dug according to the standard of 1 m × 1 m × 1 m. Using the ring-knife method, ring-knives were inserted into 5 layers of the soil profile: 0 – 20 cm, 20 – 40 cm, 40 – 60 cm, 60 – 80 cm, and 80 – 100 cm. Three 100 cm³ ring-knives were inserted into each layer. After taking out the ring-knives, they were wrapped with plastic wrap and promptly taken back to the laboratory and stored in a 4°C refrigerator.

#### 2.2.3 Biomass and carbon content determination

(1) Determination of Biomass and Carbon Content in the Tree Layer:

Trunk Biomass: A tree core of the standard tree was drilled at 1.3 m of the trunk with an increment borer. The volume of the fresh sample of the tree core was measured by the water-displacement method, and then it was placed in an 80°C oven and dried to a constant weight to measure its dry weight. Finally, the basic density of the trunk was obtained by dividing the dry weight of the sample by the volume of the fresh sample. For the tree biomass model and carbon measurement of standing poplar trees, the national standard of the People’s Republic of China *Biomass Models and Carbon Measurement Parameters of Standing Trees of Major Tree Species* (GB/T 43648-2024) was implemented. A binary stem volume model, which is primarily designed for calculating the volume of major tree species in northern China, was adopted to determine the stem volume. The model is expressed as follows (Equation 1).

(1) V=0.00005599×DBH1.89521×H0.94759

In this model, V represents the stem volume with the unit of cubic meters; DBH denotes the diameter at breast height with the unit of centimeters, and the applicable threshold of the model is set as DBH ≥ 5.0 cm; H refers to the tree height with the unit of meters.

Based on the basic density of the stem, the fresh volume of the stem was converted into the dry weight of the stem, namely the stem biomass (Fortier et al., 2017). Subsequently, the oven-dried tree core samples were grounded into powder and passed through a 100-mesh sieve. The carbon content of the processed samples was then determined by the potassium dichromate external heating method.

Branch Biomass: The branches were sawed off according to thick branches (≥ 10 cm), medium-sized branches (5 – 10 cm), thin branches (3 – 5 cm), and small branches (≤ 0.5 cm), and the mass of each standard branch was recorded. Three standard branches of each type were selected, and their fresh weights were measured. The volume of each type of standard branch was measured by the water-displacement method, and then they were placed in an 80 °C oven and dried to a constant weight. The basic densities of thick branches, medium-sized branches, thin branches, and small branches were calculated respectively, which was the ratio of dry mass to fresh sample volume. The biomass was converted by multiplying the basic density and volume of the standard branch. The standard branches of thick branches, medium-sized branches, thin branches, and small branches were grounded and passed through a 100-mesh sieve, and their carbon contents were determined by the potassium dichromate external heating method.

Leaf Biomass: The leaves stripped from the above - mentioned standard branches were weighed for their fresh weight. In the laboratory, they were placed in an 80 °C oven and dried to a constant weight to calculate the water content of the leaves, so as to calculate the total dry weight of the leaves. The standard leaves were dried, grounded, and passed through a 100-mesh sieve, and their carbon content was determined by the potassium dichromate external heating method. In summary, the sum of the biomasses of the trunk, branches, and leaves was the total above-ground biomass.

Root Biomass: In the laboratory, the soil on the surface of the roots was washed away with running water, and then the moisture on the surface of the roots was wiped dry with absorbent paper. Dead roots and live roots were classified according to the texture, color, smell of the roots, and the ease of root bark separation (Jagodziński and Kałucka, 2011). The roots were divided into fine roots (φ ≤ 2 mm) and coarse roots (φ > 2 mm) according to their diameters. The washed coarse roots, fine roots, and dead roots were weighed on an electronic balance (accuracy 0.001 g) for their fresh weights and then placed in an 80°C oven and dried to a constant weight to weigh their dry weights. Finally, the coarse roots, fine roots, and dead roots were respectively grounded and passed through a 100-mesh sieve, and their carbon contents were determined by the potassium dichromate external heating method.

The calculation formulas for the biomasses of coarse roots, fine roots, and dead roots are as follows (Equations 2, 3) (Sun et al., 2021):

(2) $$B=m/[\pi {\left(d/2\right)}^{2}]\times 10^{4}$$

(3) $$B_{singletree}=B/N\times 10^{3}$$

Where *B* is the biomass of coarse roots, fine roots, and dead roots (g·m^2^), *d* is the diameter of the soil auger (cm), *m* is the dry mass of coarse roots, fine roots, and dead roots in the soil auger (g), *B_singletree_* is the biomass of coarse roots, fine roots, and dead roots per single tree (kg·tree^-1^), and *N* is the stand density (trees·m^2^).

Total Biomass of the Tree Layer: The total biomass of each organ was added up to obtain the total biomass per single tree (Equations 1–3).

(2) Determination of Carbon Content and Environmental Factors in the Soil Layer:

Layers of 0 – 20 cm, 20 – 40 cm, 40 – 60 cm, 60 – 80 cm, and 80 – 100 cm were weighed for their fresh weights respectively, and then placed in an 80°C oven and dried to a constant weight to weigh their dry masses (accuracy 0.0001 g). Then, the soil water content (SWC), soil bulk density (SBD), minimum water - holding capacity (FC), and porosity (STP) and other physical properties were measured by the ring-knife method (Bao, 2000). The soil total carbon (TC) and soil organic matter (SOM)was determined by the potassium dichromate external heating method, the soil total nitrogen (TN) content was determined by the Kjeldahl method, the soil total phosphorus (TP) content was determined by the molybdenum-antimony-resistance colorimetric method, and the soil available potassium (AK) content was determined by the flame photometer method (Bao, 2000). The soil pH value and electrical conductivity (EC) were measured by a pH meter and an electrical conductivity meter respectively.

#### 2.2.4 Carbon storage calculation

(1) Calculation of Carbon Storage in the Tree Layer (Equation 4) (Jiang, 2023):

(4) $$C\;=\;\Sigma_{i=1}^{n}B_{i}\times C_{i}/100$$

Where *C* is the carbon storage of the tree layer, with the unit of *t*·*hm*^−2^; *B_i_* is the biomass per unit area of each component in the tree layer, with the unit of kg·m^2^; *C_i_* is the carbon content of each component in the tree layer, with the unit of *g*·*kg*^−1^.

(2) Calculation of Carbon Storage in the Soil Layer (Equation 5) (Jiang, 2023):

(5) $$SOC\;=\;\Sigma_{i=1}^{n}C_{\text{SO}{,}_{i}}\times D_{i}\times T_{i}/10$$

Where *SOC* is the soil organic carbon storage, with the unit of *t*·*hm*^−2^; *CSO_i_* is the soil organic carbon content of the *i*-th layer, with the unit of g·kg^-1^; *D_i_* is the soil bulk density of the *i*-th layer, with the unit of g·cm^-3^; *T_i_* is the depth of the soil profile of the *i*-th layer, with the unit of cm.

### 2.3 Data processing and analysis

Data were organized using Microsoft Excel 2021. Statistical analysis and significance tests were carried out using SPSS 27.0 software. One-way analysis of variance (ANOVA) and the least significant difference method (LSD) were used to compare the differences in carbon storage of the trunk, branches, leaves, roots, and soil and their influencing factors among *Populus* planted forests of different ages. Origin 2021 was used for the linear fitting of the carbon storage of *Populus* planted forests to analyze the linear relationship between the data. The Pearson correlation analysis method was used to analyze the correlation between environmental factors and the carbon storage of *Populus* planted forests (two-tailed test). Principal component analysis (PCA) and partial least squares structural equation modeling (PLS-SEM) were used to further analyze the correlation between environmental factors and the carbon storage of the trunk, branches, leaves, roots, and soil of *Populus* planted forests to find out the key environmental factors. Origin 2021 was used to draw relevant charts.

## 3 Results

### 3.1 Distribution characteristics of carbon storage in the trunk, branches, leaves, and roots of *Populus* planted forests of different ages

**Figure 2:**
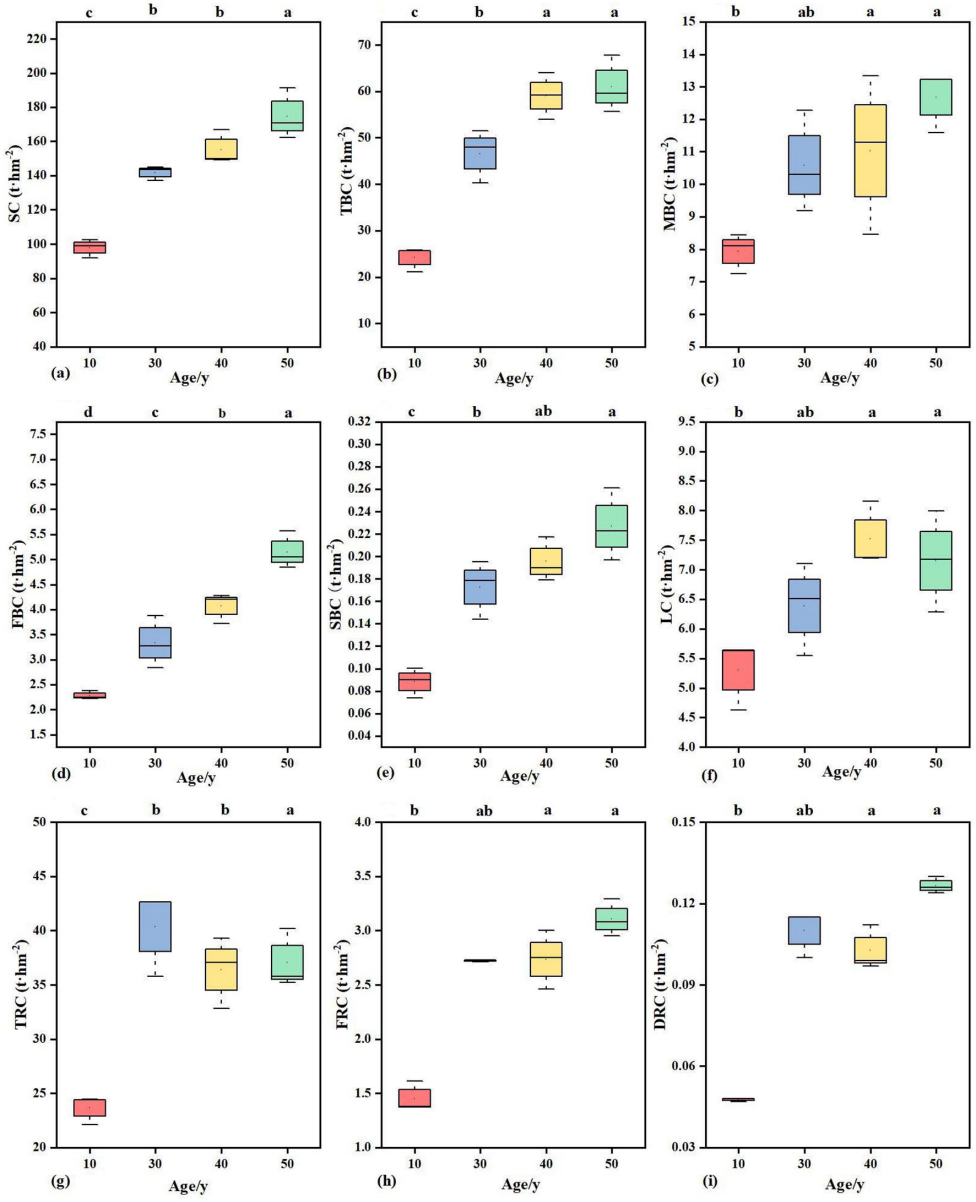
Distribution of carbon storage in different organs of populus plantations with different stand ages. Different lowercase letters (a, b, c) indicated significant differences in carbon storage of the same organ in different stand ages (*P*<0.05); **(a)** SC, Stem carbon storage; **(b)** TBC, Thick-branch carbon storage; **(c)** MBC, Medium-branch carbon storage; **(d)** FBC, Fine-branch carbon storage; **(e)** SBC, Small-branch carbon storage; **(f)** LC, Leaf carbon storage; **(g)** TRC, Thick-root carbon storage; **(h)** FRC, Fine-root carbon storage; **(i)** DRC, Dead-root carbon storage.

In *Populus* plantations of different stand ages, the carbon storage in trunks, branches, and leaves showed a gradual increasing trend with increasing stand age (Equation 4, **Figures 2a–f**). Among all age groups, the trunk carbon storage differed significantly (*P* < 0.05), with the minimum value observed at 10 y (97.75 t·hm^2^) and the maximum at 50 y (174.73 t·hm^2^). For branch carbon storage, the smallest carbon storage in small branches was recorded at 10 y (0.09 t·hm^2^), while the largest carbon storage in thick branches was observed at 50 y (60.99 t·hm^2^). Stand age had a significant effect on the carbon storage of thick, medium, thin, and small branches (*P* < 0.05). Specifically, there was no significant difference in carbon storage between thick, thin, and small branches at 40 – 50 y (*P* > 0.05), but significant differences were found compared with those at 10 – 30 y (*P* < 0.05). In contrast, the carbon storage of medium branches differed significantly across all stand ages (*P* < 0.05). Leaf carbon storage was the lowest at 10 y (5.3 t·hm^2^) and peaked at 40 y (7.52 t·hm^2^), with significant differences between 10 y and 30 - 50 y (P < 0.05). Variations in root carbon storage were observed among *Populus* plantations of different stand ages (**Figures 2g–i**). The carbon storage of coarse roots was the smallest at 10 y (23.65 t·hm^2^) and the largest at 30 y (40.35 t·hm^2^); that of fine roots was the smallest at 10 y (1.45 t·hm^2^) and the largest at 50 y (3.11 t·hm^2^); and that of dead roots was the smallest at 10 y (0.06 t·hm^2^) and the largest at 50 y (0.13 t·hm^2^).

### 3.2 Distribution characteristics of soil carbon storage in *Populus* planted forests of different ages

**Figure 3:**
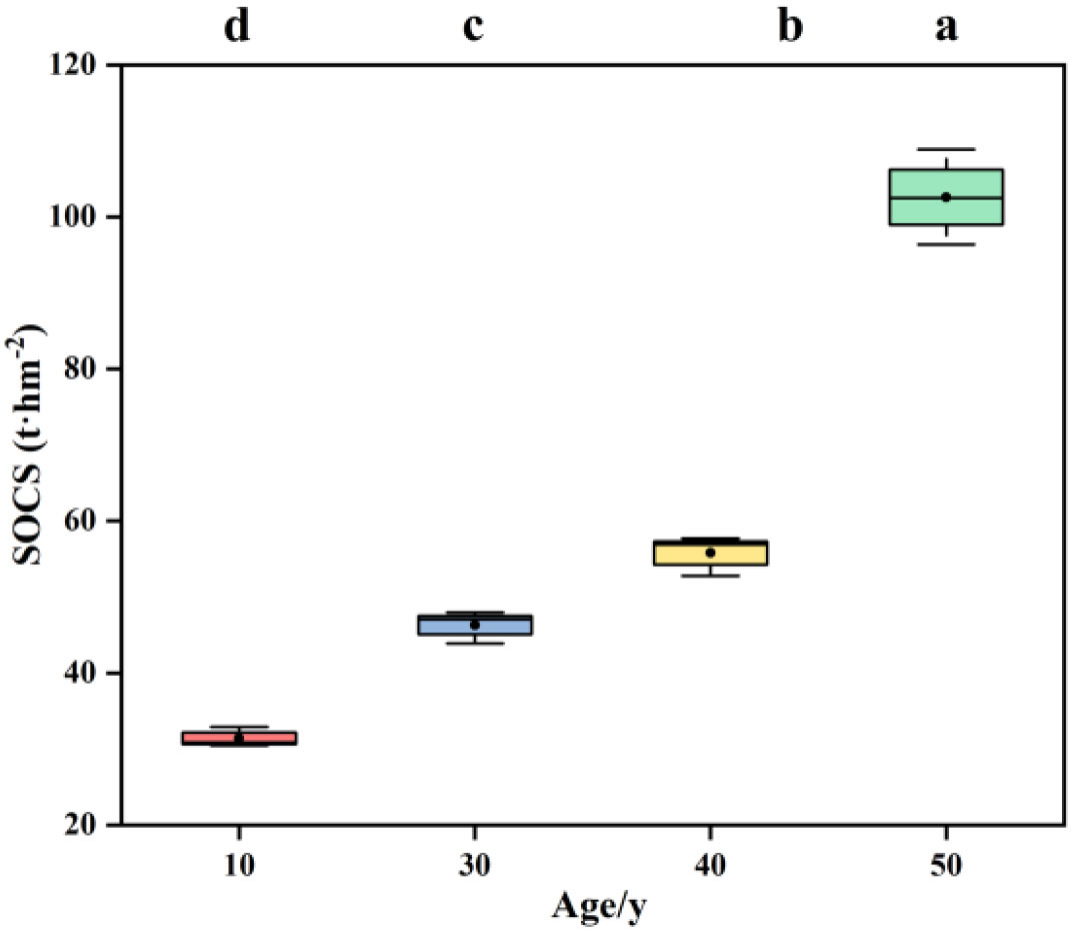
Soil layer carbon storage in *populus* plantations with different stand ages. SOCS, Soil organic carbon storage.

The soil carbon storage in *Populus* plantations of different stand ages gradually increased with increasing stand age, with significant differences observed among different age groups (Equation 5, **Figure 3**). Specifically, the soil carbon storage was the lowest at 10 y (31.42 t·hm^2^) and the highest at 50 y (102.57 t·hm^2^). Overall, the order of soil carbon storage across different stand ages was as follows: 50 y > 40 y > 30 y > 10 y.

### 3.3 Total carbon storage of *Populus* planted forests of different ages

**Figure 4:**
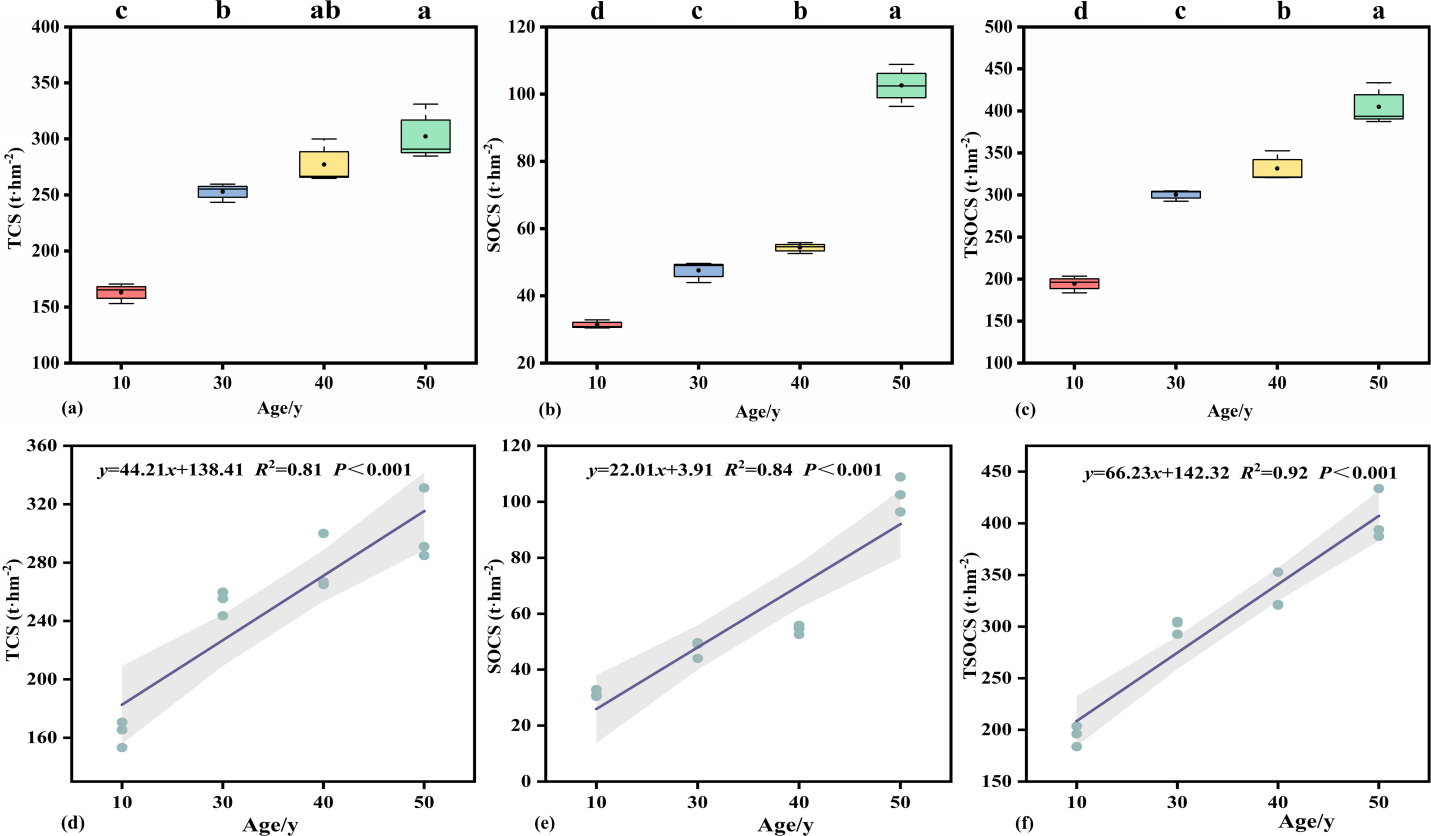
The relationship between different forest ages and carbon storage in *populus* planted forests. Different lowercase letters (a, b, c) indicate significant differences in carbon storage at the same level at different forest ages (*P*<0.05);TCS, **(a, c)** TCS, Tree layer carbon storage; **(b, e)** SOCS, Soil organic carbon storage; **(c, f)** TSOCS, Plantation forests total carbon storage.

The carbon storage of the arbor layer, soil layer and total carbon storage of poplar plantations with different stand ages all exhibited an increasing trend with the extension of stand age. Significant differences in the carbon storage of both the arbor layer and soil layer were observed among poplar plantations of different stand ages (*P* < 0.05, **Table 3**, **Figures 4a–c**). As the primary carbon pool, the arbor layer accounts for 74.67% to 84.52% of the total carbon storage in *Populus* plantations, followed by the soil layer, which contributes 15.48% to 25.33% and shows a gradual increase in proportion with advancing stand age. In terms of total carbon storage, the minimum value in *Populus* plantations was recorded at 10 y (194.71 t·hm^2^), and the maximum at 50 y (404.93 t·hm^2^). The linear fitting results showed that the *R*^2^ values of the linear analysis of the carbon storage of the arbor layer and soil layer of *Populus* planted forests were 0.81 and 0.84 respectively, and the *R*^2^ value of the linear analysis of the total carbon storage of *Populus* planted forests was 0.94, indicating a good fitting effect. Based on the regression equation, the growth rates of promoting the carbon storage of each layer of *Populus* planted forests were 44.21% and 22.01% respectively, and the growth rate of the total carbon storage was 66.23% (**Figures 4d–f**).

**Table 3:** Carbon storage of *populus* planted forests with different ages.

Layer	Age/a			
	10 y	30 y	40 y	50 y
TCS/(t·hm^-2^)	163.13 ± 8.93c	252.93 ± 8.40b	277.22 ± 19.76ab	302.37 ± 25.115a
SOCS/(t·hm^-2^)	31.59 ± 1.72d	46.32 ± 2.84c	55.76 ± 4.19b	102.57 ± 7.96a
TSOCS/(t·hm^-2^)	194.71 ± 9.87d	299.24 ± 7.47c	333.07 ± 17.13b	404.93 ± 25.04a

Different lowercase letters indicate significant differences in carbon storage at the same level at different forest ages (*P*<0.05); TCS, Tree layer carbon storage; SOCS, Soil organic carbon storage; TSOCS, Plantation forests total carbon storage.

### 3.4 The coupling relationship between the carbon storage of the trunk, branches, leaves, roots, and soil of *Populus* planted forests of different ages and environmental factors

**Figure 5:**
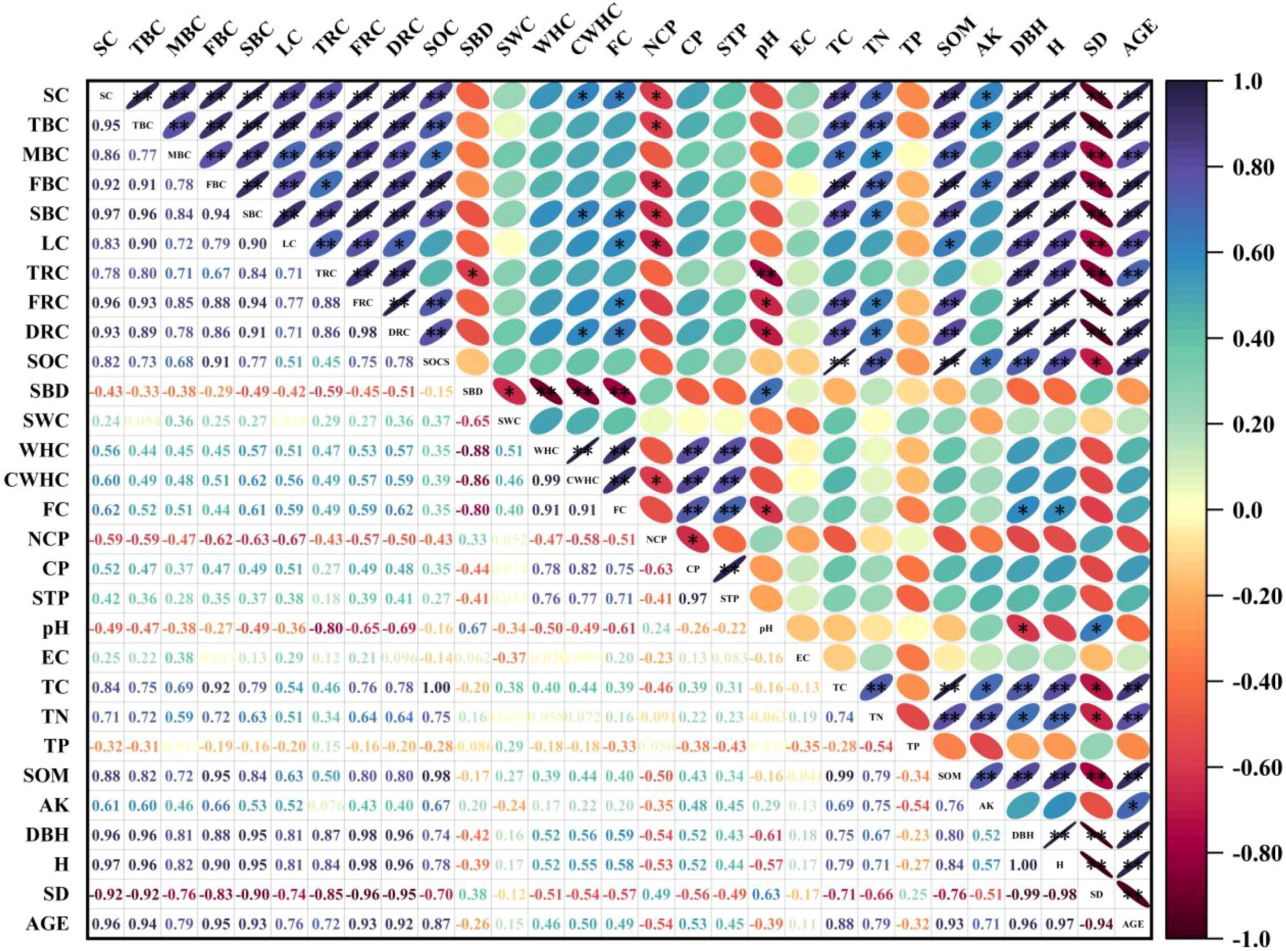
Correlations between the carbon storage of trunks, branches, leaves, roots and soil in *populus* planted forests and environmental factors. * denotes *P*<0.05, ** denotes *P*<0.01. SC, Stem carbon storage; TBC, Thick-branch carbon storage; MBC, Medium-branch carbon storage; FBC, Fine-branch carbon storage; SBC, Small-branch carbon storage; LC, Leaf carbon storage; TRC, Thick-root carbon storage; FRC, Fine-root carbon storage; DRC, Dead-root carbon storage; SOC, Soil organic carbon; SBD, Soil bulk density; SWC, Soil water content; WHC, Water Holding Capacity; CWHC, Capillary Water Holding Capacity; FC, Field Capacity; NCP, non-capillary porosity; CP, capillary porosity; STP, Total Porosity; pH, soil percentage hydrogen; EC, Electrical conductivity; TC, Total carbon; TN, Total nitrogen; TP, Total phosphorus; SOM, Soil organic matter; AK, Available potassium; DBH, Diameter at breast height; H, Height; SD, Stand density; AGE, Tree age. The same as below.

Correlation analysis indicated that the carbon storage of stems, branches, leaves, roots and soil in poplar plantations was closely associated with environmental factors (**Figure 5**). Specifically, the carbon storage of stems, branches, leaves and roots was extremely significantly positively correlated with total carbon (TC), total nitrogen (TN) and soil organic matter (SOM) (*P* < 0.01), while a significant negative correlation was found between the above carbon storage and non-capillary porosity (NCP) (*P* < 0.05). In addition, the carbon storage of stems, branches, leaves and roots exhibited extremely significant positive correlations with stand factors including diameter at breast height (DBH), tree height (H) and stand age (AGE) (*P* < 0.01), and an extremely significant negative correlation with stand density (SD) (P < 0.01). Soil organic carbon (SOC) was extremely significantly positively correlated with TC, TN, SOM, DBH, H and AGE (*P* < 0.01), whereas significant negative correlations were detected between SOCS and available potassium (AK) as well as SD (*P* < 0.05). Overall, the carbon storage of stems, branches, leaves, roots and soil showed substantial correlations with TC, TN, SOM and stand factors including DBH, H and AGE.

**Figure 6:**
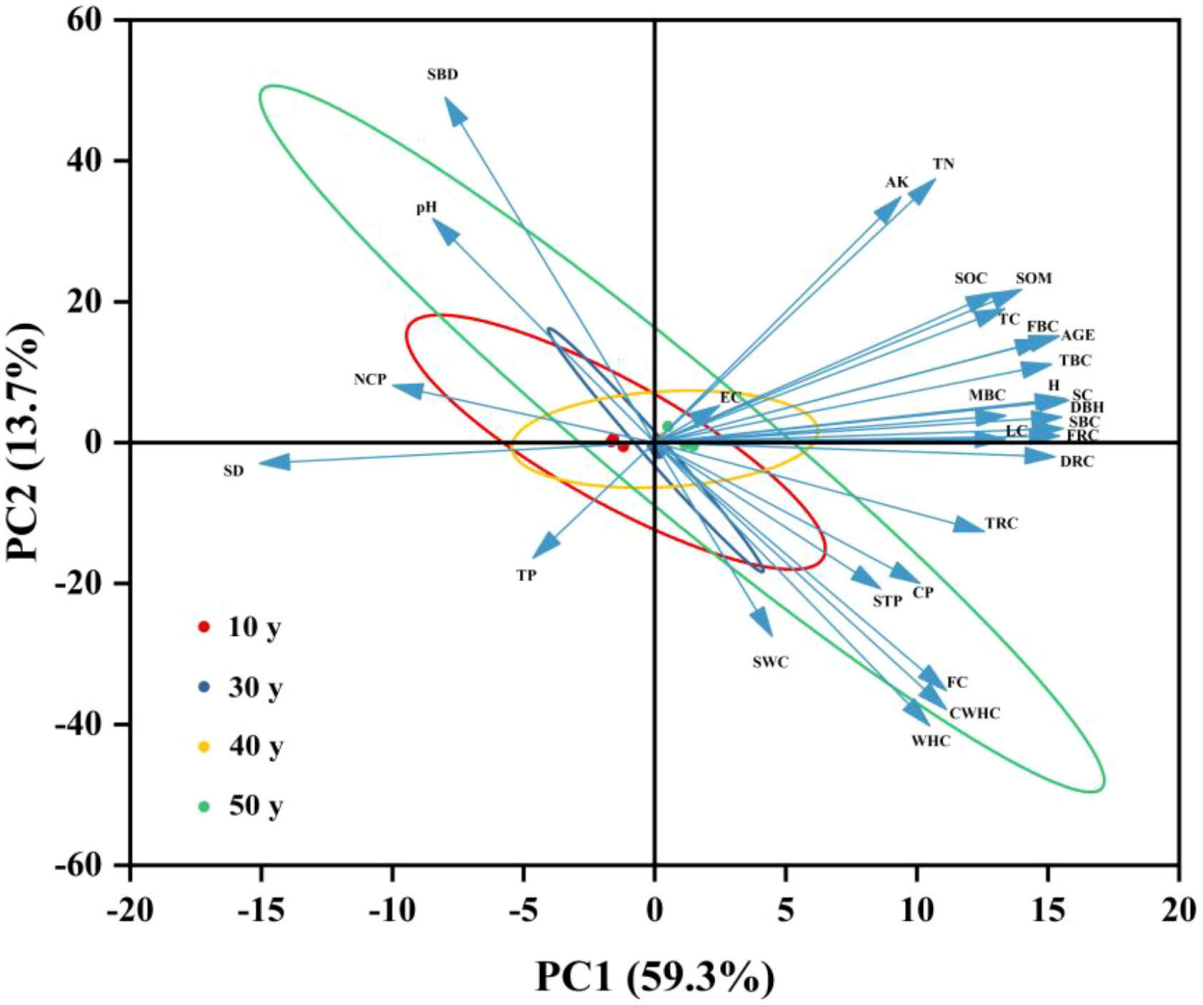
Principal component analysis of the carbon storage of trunks, branches, leaves, roots and soil in *populus* planted forests and environmental factors.

The results of principal component analysis showed that in the first principal component (PCA1), with an explanatory rate of 59.3%, it mainly reflected the main change trends of the carbon storage of the trunk, branches, leaves, roots, and soil and stand factors (**Figure 6**). In the second principal component (PCA2), with an explanatory rate of 13.7%, it was mainly related to soil physical and chemical properties. The cumulative variance contribution rate of these two principal components was 73%. The first principal component (PCA1) had a significant negative correlation with pH, SBD, SD, NCP, and TP, and a significant positive correlation with other factors. The second principal component (PCA2) had a significant negative correlation with dead-root carbon storage (DRC), thick-root carbon storage (TRC), CWHC, STP, CP, SWC, WHC, FC, TP, and SD, and a significant positive correlation with other factors. The main contributing variables of the first principal component (PCA1) were SC, TBC, SBC, SOM, DBH, H, and SD, and the main contributing variables of the second principal component (PCA2) were SBD, TN, WHC, and AK.

**Figure 7:**
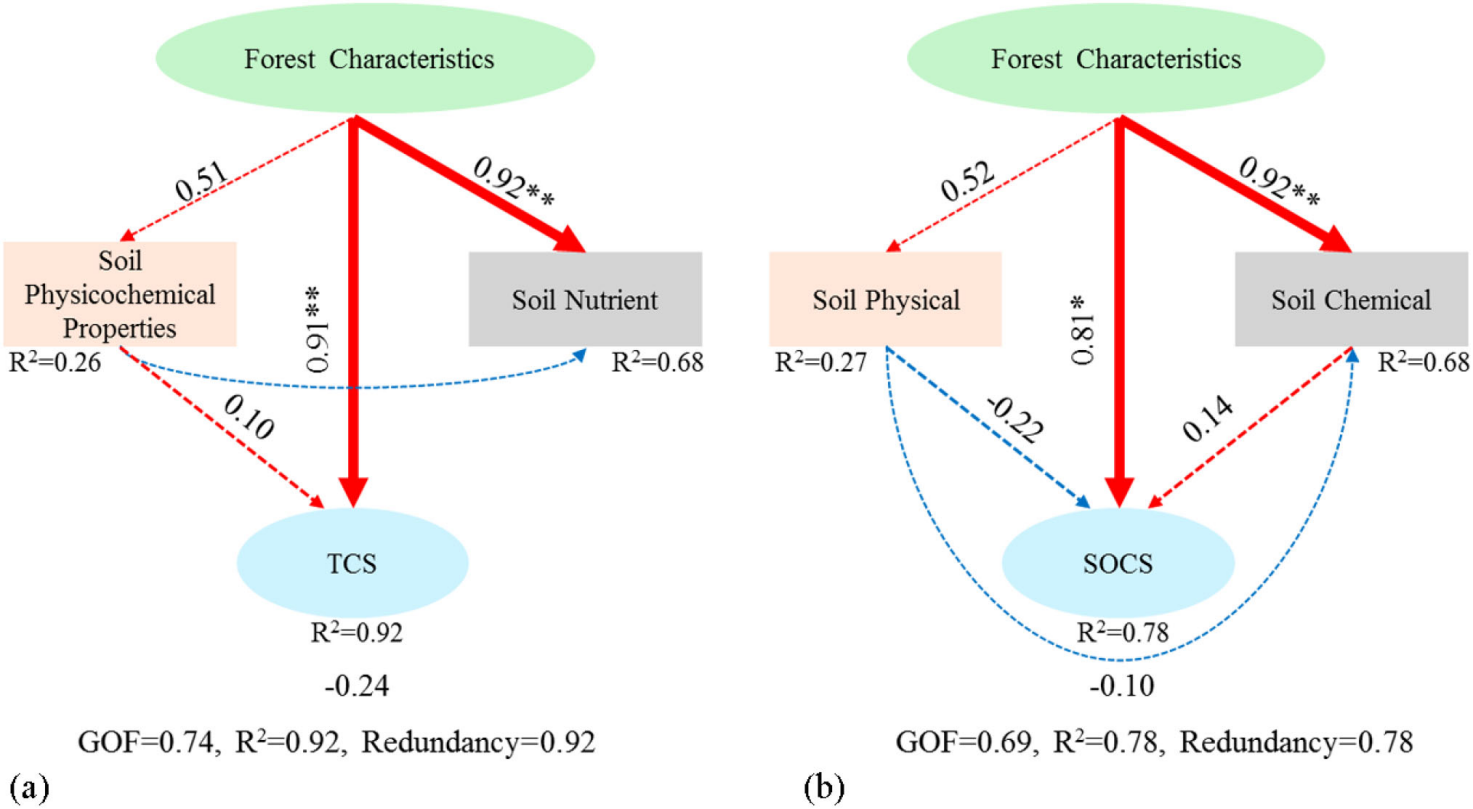
Structural equation model of the relationships between environmental factors and carbon storage of poplar plantations of *Populus* planted forests. **(a)** represents the PLS-SEM constructed with tree carbon storage as the dependent variable; **(b)** represents the PLS-SEM constructed with soil carbon storage as the dependent variable. Single arrows indicate the assumed direction of causal relationships. Red arrows indicate negative effects; blue arrows indicate positive effects. The numbers beside the arrows are standardized path coefficients. Solid arrows represent significant paths; dashed arrows represent non-significant paths. Thicker arrows indicate greater effects. *P < 0.05; **P < 0.01. R² represents the proportion of variable variance explained. TCS refers to tree carbon storage; SOCS refers to soil organic carbon storage. In model **(a)** Forest characteristics are constructed with four variables including Age, DBH, H and SD; Soil Physicochemical Properties are constructed with four soil variables including WHC, CWHC, FC and STP; Soil Nutrient are constructed with two soil variables including TN and AK contents. In model **(b)** Soil Physical are constructed with six variables including SBD, WHC, CWHC, FC, CP and STP; Soil Chemical are constructed with two soil variables including TN and AK contents.

The results of partial least squares structural equation modeling (PLS-SEM) demonstrated that stand characteristics were the most critical factor directly driving the increase in tree carbon storage per unit area (loading = 0.92, P < 0.01) and the increment of soil carbon pool under forests (loading = 0.81, P < 0.05) (**Figure 7a**). Meanwhile, stand characteristics also served as the primary factor facilitating the elevation of soil nutrient contents (**Figure 7b**).

## 4 Discussion

### 4.1 Differences in carbon storage among the trunk, branches, leaves, roots, and soil of *Populus* planted forests of different ages

Forest carbon storage is a crucial indicator for evaluating the functions of forest ecosystems (Cheng et al., 2023). Research shows that factors such as forest composition, stand density, forest age structure, and human activities have important impacts on the carbon storage of ecosystems (Justine et al., 2017; Islam et al., 2023). This study found that the total carbon storage of *Populus* planted forests of different ages showed an increasing trend with the growth of forest age, which was consistent with previous studies (Chen et al., 2019; Zhang et al., 2019; Xiang et al., 2022; Zhong et al., 2025). Among them, the total carbon storage of 50-year-old *Populus* planted forests was the highest (404.93 t·hm^2^), which was similar to the carbon storage of mangrove forests with a forest age greater than 40 y (350.53 t·hm^2^ - 483.64 t·hm^2^) in the central coastal area of Bangladesh, but lower than the carbon storage of 25.5 year-old mangrove planted forest ecosystems (190.1 t·hm^2^) (Uddin et al., 2023). This phenomenon is mainly due to the accumulation of tree biomass brought about by the increase of forest age, especially the significant increase in the biomass of the arbor layer, which improves the carbon storage of planted forests. The differences in the above-mentioned research results may be due to the impacts of climate conditions, soil types, and environmental factors on tree growth and carbon storage (Justine et al., 2017; Xiang et al., 2022; Zakaria et al., 2023).

The results of this study show that the arbor layer is the main carbon pool of *Populus* planted forests, accounting for 74.67% - 84.52% of the total carbon storage. This is because the woody organs such as the trunk and branches of *Populus* account for more than 80% of the biomass, and have high cellulose and lignin contents, strong metabolic inertia, and a long carbon sequestration cycle (Li et al., 2022). The carbon storage of the arbor layer (trunk, branches, leaves, roots) showed an increasing trend with the increase of forest age. This phenomenon may be due to the combined effect of the rapid biomass accumulation of *Populus* as a fast-growing tree species and its specific carbon allocation strategy, which promotes the increase of carbon storage in each organ of the arbor layer (Justine et al., 2017; Zakaria et al., 2023). Among them, the carbon storage of the trunk (174.73 t·hm^2^) and thick branches (60.99 t·hm^2^) both reached their maximum values at 50 y, while the carbon storage of leaves reached its maximum (7.52 t·hm^2^) at 40 y. The carbon storage of *Populus* roots was mainly distributed in the 0 – 10 cm surface layer, and the carbon storage of thick roots reached its maximum (6.28 t·hm^2^) at 50 y, which was consistent with the research of previous scholars (Justine et al., 2017; Li et al., 2018; Zhang et al., 2019). This may be because the trunk and thick branches, as the main supporting and conducting structures, due to the preferential allocation of photosynthetic products and continuous secondary growth, the carbon accumulation of trees is intensified, and the degree of lignification reaches the maximum at 50 y. The carbon storage of leaves reached its maximum during the stand-closing period at 40 y, and then decreased due to increased canopy competition leading to leaf self-thinning and resource transfer to maintain metabolism (Wang et al., 2013; Li et al., 2018). The rich nutrients and good aeration in the surface soil promote carbon accumulation, and the high microbial activity further promotes the decomposition and transformation of organic carbon, thus increasing the soil carbon storage (Truax et al., 2018; Wang et al., 2023, 2024). Soil carbon storage plays a key role in the carbon storage and carbon cycle of *Populus* planted forests (Zakaria et al., 2023). This study found that the soil carbon storage accounted for 15.48% - 25.33% of the total carbon storage of planted forests, and the proportion gradually increased with the increase of forest age, which was inconsistent with the research of Zhang et al. (2014) and Zakaria et al. (2023). This difference may be jointly caused by tree-species differences, stand density, spatial heterogeneity of soil carbon storage, geographical location, and environmental limitations (Truax et al., 2018; Meng et al., 2021). The soil carbon storage showed an upward trend with the increase of forest age and gradually decreased with the increase of soil depth. The carbon storage in the 0 – 20 cm soil layer at 50 y reached the highest value (24.13 t·hm^2^), and this trend was similar to the research of Zhang et al. (2014). In *Populus* planted forests of different ages, the carbon storage of the surface soil (0 – 40 cm) accounted for 49.17% - 69.80% of the total soil carbon storage. This is because there is more accumulation of litter and roots in the surface soil, and the sufficient oxygen supply jointly promotes carbon accumulation, thus showing a significant surface - carbon - enrichment effect (Zhang et al., 2014; Truax et al., 2018).

### 4.2 The coupling relationship between the carbon storage of the trunk, branches, leaves, roots, and soil of *Populus* planted forests of different ages and environmental factors

In this study, significant correlations were observed between the carbon storage of *Populus* plantations with different stand ages and stand factors, as well as soil factors. This indicates that stand conditions and soil nutrients play important regulatory roles in the carbon storage of *Populus* plantations in the Luxi Yellow River floodplain. Through correlation analysis, it was found that the carbon storage of each component (trunk, branches, leaves, roots) of *Populus* planted forests and the soil layer had an extremely significant positive correlation (*P* < 0.01) with stand factors (DBH and H) and soil factors (TC, TN, and SOM), and an extremely significant negative correlation (*P* < 0.01) with stand factor (SD). However, there was no significant correlation (*P* > 0.05) between carbon storage and physical and chemical properties such as SBD, SWC, WHC, CP, STP, EC, and TP. The results of this study were similar to the correlation between the carbon storage of mangrove planted forests in Malaysia and environmental factors (Zakaria et al., 2023), but different from the influencing factors of carbon storage in planted forests on the Loess Plateau studied by Li et al. (2024), which revealed the significant impacts of soil moisture, organic carbon, total nitrogen, and total phosphorus on carbon storage. This regional difference may be due to the following mechanisms: First, unlike the growth characteristics of the studied trees, some tree species are nitrogen-fixing species that can improve the soil environment in the rhizosphere by themselves. Although certain tree species are all categorized as arbor trees, differences exist in the branching structures of their trunks and crowns, which lead to variations in tree biomass. Biomass is directly correlated with tree carbon storage, and the accumulation of such carbon storage is directly affected by tree characteristics including DBH and H. Second, soil factors indirectly affect the carbon cycle process by regulating root development and the rate of organic matter mineralization (Zakaria et al., 2023). The study further revealed that the negative correlation between stand density (SD) and carbon storage may be due to the resource - competition effect caused by high-density stands. High stand density will directly lead to intensified competition for light, water, and nutrients, inhibit individual growth, and change the soil micro-environment, ultimately reducing the forest carbon storage per unit area (Li et al., 2024). The lack of correlation between soil physical and chemical properties such as SBD, SWC, WHC, CP, STP, EC, and TP and forest carbon storage may be attributed to the stable hydro-thermal conditions in the study area, the strong adaptability of *Populus* roots to the soil environment, and the weak indirect regulatory effect of these factors on carbon storage (Meng et al., 2021; Truax et al., 2018). The differences between the conclusions of this study and the previous research results on forest carbon storage may be due to differences in environmental conditions, tree-species characteristics, stand structures, and management measures in different regions, which highlights the impacts of biological and abiotic factors on forest carbon storage (Islam et al., 2023; Wang et al., 2013).

This study revealed the key driving mechanism of forest carbon storage through principal component analysis (PCA) and structural equation modeling (SEM). The PCA results showed that the cumulative variance contribution rate of these two principal components was 73.00%, mainly representing the change trends of carbon storage and stand factors (DBH, H, and AGE). This finding was consistent with the research of Zakaria et al. (2023), indicating that the growth of DBH and H directly promotes the increase of ecosystem carbon storage through biomass accumulation. The SEM path analysis further clarified the synergistic action mechanism of multiple factors: tree - growth indicators (DBH, H, forest age), forest - ecosystem characteristics (SD), soil-moisture characteristics (SWC, WHC, CWHC, FC), and soil - structure indicators (SBD, NCP, CP, STP) all had a positive impact on the carbon storage of the arbor layer and *Populus* planted forests, which was similar to the results of this study by Wang et al. (2023). This phenomenon may be due to the fact that the forest-canopy coverage promotes the photosynthesis and carbon fixation of forests, prompting tree growth to increase biomass and carbon storage. At the same time, sufficient water supply can also promote tree growth and carbon fixation. In addition, soil - structure indicators improve the aeration and water permeability of the soil, promoting the growth and carbon fixation of tree roots (Meng et al., 2023). Soil nutrients (TC, TN, TP, SOM, AK) and soil-environment chemical indicators (pH, EC) had a negative impact on carbon storage, possibly because increased microbial activity accelerated the decomposition of organic matter, thus reducing carbon accumulation (Wang et al., 2023). Stand characteristics, tree-growth indicators, and soil nutrients all had a positive impact on soil-layer carbon storage. This is mainly because forest-ecosystem characteristics and tree-growth indicators affect the input and decomposition of plant residues, thereby affecting soil carbon storage. High-content soil nutrients are conducive to the decomposition of plant residues and the fixation of plant carbon (Liu et al., 2024; Wang et al., 2024). Soil moisture, soil-structure indicators, and soil-environment chemical indicators had a negative impact on soil-layer carbon storage. This is because excessive moisture and specific soil structures may accelerate carbon decomposition, and changes in soil-environment chemical indicators may be unfavorable for carbon fixation (Hao et al., 2025). The results of this study show that H, DBH, forest age, and SD are the key factors driving forest carbon storage. These environmental factors play a key role in the carbon cycle and tree growth by affecting the growth and competition relationships of trees. In addition, the forest age determines the growth characteristics and carbon-absorption efficiency of forests, thereby affecting the carbon sequestration of plants (Zakaria et al., 2023). Due to the high randomness and spatial heterogeneity in the distribution of tree root systems, the whole-excavation method was not adopted for root sampling in this study. Instead, a soil auger was used, which may result in a smaller quantity of collected roots compared with the actual amount. Consequently, there might be a certain gap between the root data calculated by the model and the real data. In addition, the whole-plant harvesting method was not applied for collecting above-ground parts of trees, such as trunks and branches. This leads to a lower mass of collected tree samples than the actual value. Therefore, the tree biomass and carbon storage calculated by the binary stem volume model may be underestimated. However, the research results can reflect the distribution patterns of carbon storage among different stand ages to a certain extent. Furthermore, in the part of analyzing the effects of environmental factors on forest carbon storage, this study only explored the impacts of stand and soil factors, without involving topographic factors such as altitude and slope gradient. Thus, in future studies, topographic factors should be comprehensively considered to more accurately evaluate the carbon sink potential of forests, thereby providing a scientific basis for formulating forest carbon sink management strategies at the regional scale.

## 5 Conclusion

Studies on carbon storage in *Populus* plantations of different stand ages in the Luxi Yellow River floodplain revealed the following: Carbon storage in trunks, branches, and roots gradually increased with stand age, reaching maximum values at 50 y (174.73 t·hm^2^, 60.99 t·hm^2^, and 6.28 t·hm^2^, respectively). Leaf carbon storage increased with stand age, peaking at 40 y (7.52 t·hm^2^) before declining thereafter. Soil carbon storage consistently increased with stand age, with the minimum value at 10 y (0.81 t·hm^2^) and the maximum at 50 y (24.13 t·hm^2^). Additionally, soil carbon storage gradually decreased with increasing soil depth, with carbon accumulation rates in the 0 – 40 cm soil layer accounting for 49.16% to 69.77% of the total. In summary, the total carbon storage of *Populus* plantations across different age classes gradually increased with stand age. Correlation analysis, principal component analysis, and structural equation modeling indicated that DBH, tree height, age, and SD were the key factors influencing carbon storage in *Populus* plantations. These findings can provide basic data and theoretical support for formulating strategies for carbon sequestration enhancement, management, and ecological restoration of Populus plantations in the Luxi Yellow River floodplain.

## Data Availability

The original contributions presented in the study are included in the article/supplementary material. Further inquiries can be directed to the corresponding authors.
